# Temperature is a common climatic descriptor of lachryphagous activity period in *Phortica variegata* (Diptera: Drosophilidae) from multiple geographical locations

**DOI:** 10.1186/s13071-020-3955-0

**Published:** 2020-02-18

**Authors:** Marco Pombi, Valentina Marino, John Jaenike, John Graham-Brown, Ilaria Bernardini, Riccardo P. Lia, Fred Beugnet, Guadalupe Miro, Domenico Otranto

**Affiliations:** 1 Dipartimento di Sanità Pubblica e Malattie Infettive, Sapienza Università di Roma, Rome, Italy; 2 Animal Health Department, Veterinary Faculty, Universidad Complutense de Madrid, Madrid, Spain; 3 University of Rochester, Rochester, NY USA; 4 Department of Livestock Health and Welfare, Institute of Veterinary Science, University of Liverpool, Liverpool, UK; 5 Dipartimento di Medicina Veterinaria, Università degli Studi di Bari “Aldo Moro”, Bari, Italy; 6 Boehringer Ingelheim Animal Health, Lyon, France

**Keywords:** Eyeworm, Lachryphagy, Vector ecology, Vector-borne disease, Zoonosis, Environmental parameters

## Abstract

**Background:**

The drosophilid *Phortica variegata* is known as vector of *Thelazia callipaeda*, the oriental eyeworm native to Asia that has become an emergent zoonotic agent in several European regions. Unlike almost all other arthropod vectors of pathogens, only *P. variegata* males feed of lachrymal secretions of animals, ingesting first-stage larvae (L1) of the worm living in the orbital cavities of the host, and allowing with the same behaviour the introduction of infective L3. Despite the increased detection of *T. callipaeda* in many European countries, information about the length of the lachryphagous activity period of *P. variegata* and a deep knowledge of the environmental and climatic variables involved are still limited.

**Methods:**

We herein present the results of a multicentre study involving five sites from four different countries (Italy, Spain, UK and USA) where canine thelaziosis is endemic and/or where it has already been ascertained the presence of *P. variegata*. Field data have been obtained on a fortnightly basis from mid-April to the end of November 2018 from a contemporary standardized sampling (same sampling effort and time of collection in all sites) of lachryphagous flies collected around the eyes of a human bait using an entomological net. These data have been associated to data collection of local climatic variables (day length, temperature, wind speed, barometric pressure and relative humidity).

**Results:**

Overall, a total of 4862 *P. variegata* flies (4637 males and 224 females) were collected, with high differences in densities among the different sampling sites. Significant positive correlations were found between *P. variegata* male density and temperature and wind speed, while negative correlations were observed for barometric pressure and relative humidity. However, the above significant differences are confirmed in each sampling site separately only for the temperature.

**Conclusions:**

This multicentre study highlights that temperature is the major common environmental driver in describing the lachryphagous activity of *P. variegata* in Europe and USA and, therefore, the transmission risk of thelaziosis.
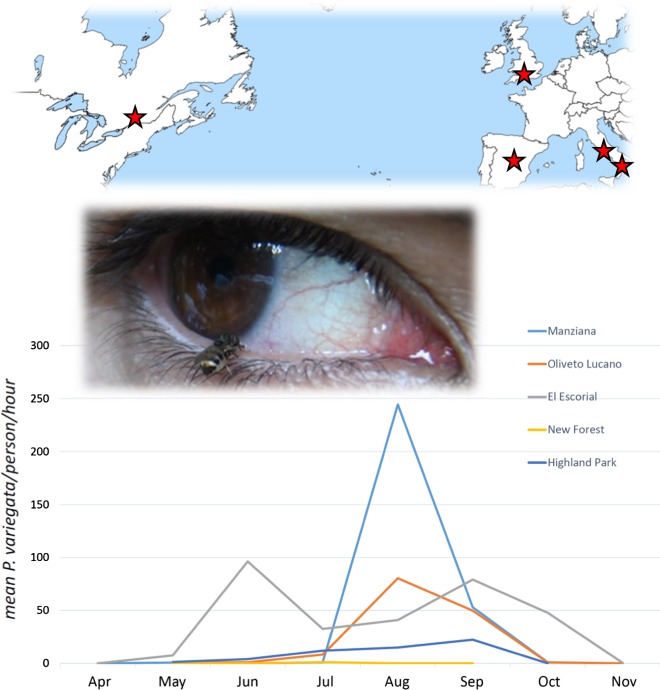

## Background

*Phortica variegata* (Diptera: Drosophilidae), a zoophilic fruit fly of the subfamily Steganinae (Drosophilidae), has attracted the interest of the scientific community because of its capacity to vector *Thelazia callipaeda* (Spirurida: Thelaziidae) eyeworms. Amongst vector-borne helminths, *T. callipaeda* is an emergent zoonotic agent of concern to the public health of several European regions. The adult nematodes live in the orbital cavities and associated host tissues of dogs, cats, foxes, wolves, rabbits and humans, causing ocular disease [1–3]. Unlike almost all other arthropod vectors of pathogens, only *P. variegata* males feed of lachrymal secretions of animals, ingesting first-stage larvae (L1) of the worm. According to data from experimental infestation [4, 5], in the vector the larval development through to infective L3 occurs within 14–21 days; larvae may also survive in overwintering flies (for up to 6 months) before being transmitted to a receptive host [4]. Although *T. callipaeda* has not been reported from the USA, *P. variegata* has been found also in this country, and it has been demonstrated that flies collected in the USA can successfully transmit the parasite [5]. At the time when vector identity and biology were elucidated, the infection was known only in remote poor settings in southern Italy [4, 6, 7]. Nevertheless, ecological niche models predicted that large areas of Europe (including the UK) are suitable for the presence of *P. variegata*, thus highlighting the potential risk of *T. callipaeda* spreading to other European countries [8, 9]. Indeed, whilst *T. callipaeda* had exclusively been reported from easternmost countries until two decades ago, it has now been described in both animals and humans from Austria, Belgium, Bosnia and Herzegovina, Bulgaria, Croatia, France, Germany, Greece, Hungary, Italy, Portugal, Romania, Serbia, Slovakia, Spain, Switzerland and Turkey [9–12]. Thus, this parasite can be considered as an emergent vector-borne pathogen in Europe, and zoonotic infections have been diagnosed on several occasions. As an example, in Spain, the first autochthonous case of thelaziosis was reported in 2010 [13]; following further reported cases of canine thelaziosis in this country, certain geographical areas are now considered endemic for this infection [14, 15]. Despite the increased detection of *T. callipaeda* in many European countries, information about the length of the lachryphagous activity period of *P. variegata* and a deep knowledge of the environmental and climatic variables involved are still limited.

We herein present the results of a multicentre study involving five sites from four different countries (Italy, Spain, UK and USA) where canine thelaziosis is endemic and/or where it has already been ascertained the presence of *P. variegata*. Data obtained from a contemporary standardized field sampling over the whole reproductive season of the vector, associated with data collection of local climatic variables, elucidated the common environmental drivers of the population dynamics of *P. variegata* in Europe and North America. These results are of major importance in describing the climatic features associated to the abundance of lachryphagous males of *P. variegata* and, when put in the context of climate change, the epidemiological consequences of the transmission of *T. callipaeda* to the vertebrate hosts, highlighting the importance of thelaziosis among the zoonosis of public health relevance that should not be neglected.

## Methods

A longitudinal sampling was performed on a fortnightly basis from the mid of April to the end of November 2018 in five areas of four different countries in which *P. variegata* presence was already recorded. Each sampling site is described below.

### Sampling sites

Highland Park (Rochester, NY, USA, 43°07′59″N, 7°36′43″W; altitude 240 m a.s.l.). This ~ 60 ha park was established in 1888 within a low-density urban area whose total population is 208,000. The park occurs on a glacial moraine with alfisol soil. The area occurs with the humid continental climate zone with substantial seasonal variation in temperature. The site within the park where flies were collected includes relatively steep-sided slopes and flatter areas between the slopes. *Quercus rubra* is the dominant tree species, with individuals up to 150 cm in diameter at breast height (dbh). Other trees at the site include *Juglans nigra*, *Prunus serotina*, *Acer saccharum*, *Gymnocladus dioicus*, *Betula papyrifera* and *Tilia americana.* There is little understory except at the margins of the wooded areas, where the shrubs *Rubus allegheniensis* and *Cercis canadensis* occur. Animals at the site include *Odocoileus virginianus*, *Vulpes vulpes*, *Sciurus carolinensis*, *Tamias striatus* and *Marmota monax*.

New Forest (Hampshire district, England, UK, 50°50′32″N, 01°30′53″W; altitude 20 m a.s.l.), a 219 square miles national park located on the south coast of mainland England. The climate is typical of the UK generally, with a temperate seasonal pattern of cold to cool winters and mild to warm summers with moderate rainfall throughout the year. The park is composed of a mixture of deciduous forest, predominantly *Quercus rubra* with *Fagus sylvatica* and *Betula pendula*, pastures and healthlands grazed by horses, cattle and wild cervids (*Capreolus capreolus* and *Dama dama*). The park is also home to a number of other sylvatic species common to british woodland habitats, including *Meles meles* and *Vulpes vulpes*. The trapping site used for this study was on a boundary between woodland and heath at a location where *P. variegata* has been documented on several occasions previously [9].

El Escorial (Sierra de Guadarrama, Madrid, Spain, 40°36′10″N, 04°07′22″W; altitude 946 m a.s.l.). “La Herrería” forest is an area of 499 ha located in the “Sierra de Guadarrama”, at 2.5 km south of the urban center of the municipality of El Escorial (northwest Madrid Community). The climate and vegetation are typically Mediterranean. Thus, summers are hot and dry, and maximum rainfall is recorded in autumn and spring. The vegetation consists of a rich range of herbaceous and woody species such as *Quercus pyrenaica*, *Ilex aquifolium*, *Betula celtiberica*, *Crataegus monogyna* and *Pinus brutia*. Animals most represented are *Cervus elaphus*, *Sus scrofa*, *Capreolus capreolus*, *Dama dama*, *Meles meles*, mustelids, *Felis silvestris*, *Vulpes vulpes*, *Canis lupus* and *Lepus europaeus*.

Manziana (Lazio region, Italy, 42°07′09″N, 12°06′58″E; altitude 378 m a.s.l.). This region is a slightly hilly forested area of 545 ha of volcanic origin with poor Mediterranean climatic characteristics. The dominant tree species is *Quercus cerris*, which is mostly associated with *Quercus frainetto*. The dominant shrub species is *Mespilus germanica*, associated with *Carpinus betulus*, *Acer monspessulanum*, *Acer campestre*, *Ilex aquifolium*, *Fraxinus ornus*, *Sorbus torminalis*, *Ruscus aculeatus*, *Ulmus minor*, *Malus sylvestris*, *Crataegus monogyna*. The most abundant wild animal species are *Vulpes vulpes*, *Sciurus vulgaris*, *Martes foina*, *Sus scrofa*, *Hystrix cristata*, *Lepus europaeus*. The forest is surrounded by a hurbanized area (7700 inhabitants) and is also frequented by domestic species such as dogs, horses and cattle.

Oliveto Lucano (Basilicata region, Italy, 40°32′37″N, 16°09′18″E; altitude 900 m a.s.l.). The Park of Gallipoli Cognato covers an area of 27.027 ha within the borders of the towns of Accettura, Calciano and Oliveto Lucano in the province of Matera, and Castelmezzano and Pietrapertosa in the province of Potenza. In the all municipalities of the park live 4900 inhabitants. It is a mountain area of arenaceous rock, with peaks above 1000 m a.s.l. (e.g. mount Caperrino 1400 m a.s.l.). The presence of watercourses is conspicuous but in the form of seasonal torrents. In the forest the tree species are *Quercus cerris*, *Quercus petraea*, *Quercus pubescens* and *Quercus frainetto*. Moreover, *Carpinus betulus*, *Tilia cordata*, *Acer campestre*, *Acer monospessullanum*, *Sorbus domestica* and *Fraxinus angustifolia* are present. The shrub layer is mainly composed of *Rubus fruticosus*, *Erica arborea*, *Genista tinctoria*, *Prumus spinosa*, *Amelanchier ovalis*, *Crataegus monogyna*, *Sambucus nigra*, and *Ilex aquifolium*. Wild animal species present are: *Vulpes vulpes*, *Martes foina*, *Martes martes*, *Mustela nivalis*, *Erinaceus europaeus*, *Hystrix cristata*, *Lepus europeaeus*, *Lepus corsicanus*, *Sus scrofa*, *Sciurus vulgaris*, *Canis lupis* and *Felis silvestris*. The forest is also frequented by domestic species such dogs, cats, sheep, pigs and cattle.

### Collection of lachryphagous flies

In each sampling area two sites 200–400 m far from each other were chosen, to take into account for the local variability in the fly distribution. The sampling effort was 30 min per site, which was carried out twice in each sampling day (11:00–12:00 h and 15:00–16:00 h). Lachryphagous flies were collected around the eyes of a human bait using an entomological net, brought to the laboratory, and morphologically identified under a stereomicroscope [16].

### Analysis of *P. variegata* abundance

*Phortica variegata* abundance obtained in each site was corrected, taking into account the effective sampling time as well as the number of collectors and baits used in each sampling (due to the occasional participation of two collectors at the same time). The daily abundance of lachryphagous males and females was transformed as log_10_ (x + 1) of flies per person per hour in order to linearize the relationship with climatic variables. These values were then independently associated to the following climatic variables, collected daily from each site (Additional file 1: Table S1): day length (expressed as % light time in 24 h), temperature (mean, minimum, maximum, expressed as °C), relative humidity (expressed as %), barometric pressure (expressed as mbar), mean wind speed (expressed as m/s) in order to detect possible correlations. Relationships between fly densities and climatic variables were calculated by Kendallʼs tau-b rank correlations as a non-parametric measure adjusted for tied ranks using Statsdirect software version 3.2.8 [17].

## Results

**Fig. 1:**
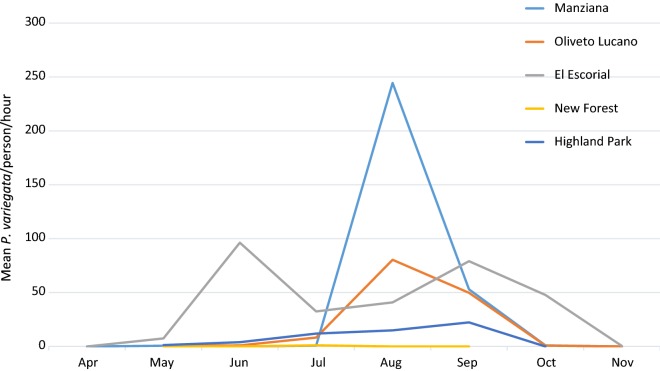
Population dynamics of *Phortica variegata* flies collected (expressed as mean numbers per person per hour) over the months of sampling in all sites

Overall, a total of 4862 *P. variegata* flies (4637 males and 224 females) were collected, with high differences in densities among the different sampling sites (Table 1). In particular, the largest collection was obtained from the sites at the lowest latitude, El Escorial (Spain), with 2387 *P. variegata* flies, followed by Manziana (Italy, *n* = 1204), Oliveto Lucano (Italy, *n* = 1127), Highland Park (USA, *n* = 135) and New Forest (England, *n* = 9). Data on mean fly densities corrected for the sampling effort (expressed as number of flies per person per hour; Table 1, Fig. 1) showed a similar picture: El Escorial (141.3); Manziana (141.6); Oliveto Lucano (75.1); Highland Park (9.0); and New Forest (0.4). Population dynamics of *P. variegata* in the different sites showed a unimodal curve with a common peak in density during weeks 34–36 (corresponding to the sampling dates 22nd August to 7th September 2018) in all sites but El Escorial. In the latter, a multimodal distribution has been observed, showing at least 4 peaks (weeks 20, 26, 36 and 40) with a maximum at week 26.

**Table 1:** Collections of Phortica variegata flies over the sampling period (expressed as month and week of the calendar number) in all sites

Month	Week	Highland Park			New Forest			El Escorial			Manziana			Oliveto Lucano		
		Mean fly/p/h	Total catch	Mean male (%)	Mean fly/p/h	Total catch	Mean male (%)	Mean fly/p/h	Total catch	Mean male (%)	Mean fly/p/h	Total catch	Mean male (%)	Mean fly/p/h	Total catch	Mean male (%)
April	16							0	0							
	17										0	0				
May	18	0	0		0	0		16	16	100	0	0		0	0	
	19										0	0				
	20	1.2	1	100	0	0		57	60	95	12	6	100	2	2	100
	21										2	1	100			
	22	5	5	100				13	14	92.9	0	0		5	5	100
June	24	3	3	100	0	0		93	94	98.9	0	0		4	4	100
	26	5	5	100	0	0		675	676	100	0	0				
July	28	12	12	100	1.7	5	100	184	184	100	6	3	100	23	23	100
	30	11	12	91.7	2.5	4	100	75	76	98.7	0	0		42	43	97.7
August	32	20	20	100	0	0		66	68	97.1	636	318	100	101	101	100
	34	8	10	80	0	0		255	259	98.5	1320	660	100	539	542	99.4
September	36	42	43	97.7	0	0		377	385	97.9	352	176	100	280	281	100
	38	22	23	95.7	0	0		84	74	93.2	72	36	100	117	117	100
	39	1	1	100												
October	40	0	0					170	254	66.9				8	9	88.9
	42	0	0					122	224	54.5	8	4	100	0	0	
	43										0	0				
	44	0	0					0	0					0	0	
November	46							1	3	33.3	0	0		0	0	
	47													0	0	
	48							0	0							
Total		9	135	96.3	0.4	9	100	141.3	2387	91	141.6	1204	100	75.1	1127	99.5

*Abbreviations*: Mean fly/p/h, mean number of flies per person per hour; total catch, total number of flies collected in the day of sampling; male %, percentage of males individual on the total of catches

**Fig. 2:**
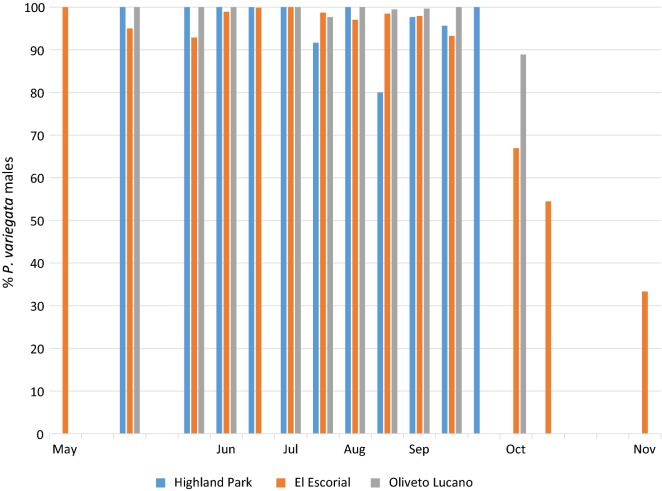
Percentage of *Phortica variegata* males collected overall weeks of sampling (in numbers). Only the sites in which female presence have been observed were here considered

The male/female ratio obtained from the collection around the eyes of the human bait was heterogeneous (Table 1, Fig. 2), with sites in which only males were collected (Manziana and New Forest), and others in which some females were also trapped: El Escorial 9%; Highland Park 3.7%; and Oliveto Lucano 0.5%. This supposed lachryphagous activity of females seems to be present all over the sampling season, without a specific pattern. However, a strong increase in female collection (> 30%) was observed from October in El Escorial.

Pooling data from all sites of the four countries, *P. variegata* male densities, expressed as the logarithm of flies per person per hour, showed significant positive correlations with temperature (maximum, minimum and mean) and mean daily wind speed, while negative correlations were observed for barometric pressure and relative humidity. However, the above significant differences are confirmed in each sampling site separately only for the temperature parameters (Table 2), with the exception of New Forest, in which no significant correlations were found. The other correlations observed in the single sites excluding the temperature were a negative correlation with wind speed in Manziana and with relative humidity in El Escorial.

**Table 2:** Kendallʼs rank correlation of climatic variables *versus* Phortica variegata males referred to the day of sampling

Site	Variable	Valid *n*	Kendall’s tau b	*P*-value
All sites	Day length	76	0.021	0.80
	*Maximum temperature*	75	0.45	< 0.0001
	*Minimum temperature*	75	0.40	< 0.0001
	*Mean temperature*	75	0.43	< 0.0001
	*Mean wind speed*	73	0.17	0.044
	*Barometric pressure*	76	− 0.25	0.003
	*Relative humidity*^a^	55	− 0.23	0.014
Manziana	Day length	17	− 0.16	0.44
	Maximum temperature	16	0.30	0.15
	*Minimum temperature*	16	0.15	0.025
	Mean temperature	16	0.34	0.10
	*Mean wind speed*	17	-0.40	0.039
	Barometric pressure	17	0.13	0.53
	Relative humidity	7	0.59	0.10
Oliveto Lucano	Day length	17	0.27	0.16
	*Maximum temperature*	17	0.58	0.002
	*Minimum temperature*	17	0.39	0.041
	*Mean temperature*	17	0.47	0.015
	Mean wind speed	17	− 0.15	0.44
	Barometric pressure	17	− 0.08	0.70
	Relative humidity	17	− 0.07	0.74
El Escorial	Day length	17	0.30	0.10
	*Maximum temperature*	17	0.57	0.002
	*Minimum temperature*	17	0.48	0.008
	*Mean temperature*	17	0.59	0.001
	Mean wind speed	14	− 0.16	0.47
	Barometric pressure	17	0.35	0.057
	*Relative humidity*	16	− 0.39	0.042
New Forest	Day length	10	0.18	0.60
	Maximum temperature	10	0.55	0.07
	Minimum temperature	10	0.22	0.51
	Mean temperature	10	0.44	0.15
	Mean wind speed	10	− 0.11	0.80
	Barometric pressure	10	− 0.11	0.80
	Relative humidity	na	na	na
Highland Park	Day length	15	0.24	0.25
	*Maximum temperature*	15	0.56	0.006
	*Minimum temperature*	15	0.76	0.0002
	*Mean temperature*	15	0.52	0.010
	Mean wind speed	15	0.06	0.80
	Barometric pressure	15	− 0.18	0.39
	Relative humidity	15	0.08	0.73

^a^New Forest data not available

*Notes*: Day length recorded as % of light in 24 hours; temperature recorded as °C; wind speed recorded as m/s; pressure recorded as hPa; humidity recorded as %. Italic highlights the variables with significant correlations (*P*-value < 0.05)

*Abbreviations*: Valid n, records used in the statistics; Kendallʼs tau b, correlation value; *P*-value, two-sided significance level of the b-statistics; na, data not available

The temperature of activity of lachryphagous males (in which at least one fly was collected) varies in different sites, ranging from a daily minimum of 7 °C (Manziana) to a daily maximum of 35 °C (El Escorial), despite the temperature range during the sampling period in all sites varied from 0 °C to 36 °C (Table 3).

**Table 3:** Temperature (°C) ranges (minimum–maximum) for which at least one male individual of *Phortica variegata* was collected and the temperature range measured over the sampling period in each site of the study

Site	Min. T (°C)	Max T (°C)	Temperature range (°C)
Highland Park	9	34	0–34
New Forest	11	29	1–29
El Escorial	8	35	3–35
Manziana	7	34	5–36
Oliveto Lucano	9	32	9–32

The correlation analysis of female densities of *P. variegata* (expressed as logarithm of flies per person per hour), cumulatively collected in the sites of Oliveto Lucano, El Escorial and Highland park, shows significant positive correlation with wind speed (Kendall’s tau *b* = 0.24; *P* = 0.043) and negative correlations with pressure (Kendall’s tau *b* = − 0.38; *P* = 0.0008) and relative humidity (Kendall’s tau *b* = − 0.29; *P* = 0.010).

## Discussion

Our data indicate that temperature is an important driver of the seasonal dynamics of *P. variegata* lachryphagous males in the countries sampled, indicating that this climatic variable in particular is directly associated with the lachryphagous behaviour and therefore with the *T. callipaeda* transmission risk.

Vector-borne diseases follow complex epidemiological patterns due to the interaction of the pathogen, the vector and the vertebrate host/s species [18, 19]. In the case of thelaziosis, the records of infected dogs, as well as other hosts including humans, are increasing in the past decade in several European countries, indicating that *T. callipaeda* is spreading continent-wide. This is probably due to the diffusion of the vector *P. variegata* or, more likely, to the movements of infected dogs in areas where the vector is present. The latter hypothesis is the most likely considering the nearly ubiquitous distribution of *Phortica* spp. flies [16]. The knowledge of the ecological niche of *P. variegata* is of major importance to understand if and how eyeworm infection can spread locally in different contexts, and what are the environmental drivers potentially favouring the establishment of the vector at a continental scale. A habitat suitability map of this species has been established, indicating a large potential of diffusion in Europe [8, 9]. However, data at the origin of the development of the model are fragmentary and, more importantly, no information is available concerning environmental parameters affecting the population dynamics of *P. variegata*, except those obtained from a few sites [8, 14]. The multicentre approach herein presented allows identification of the climatic parameters that correlate with the population dynamics of *P. variegata* across the reproductive season.

Among the five sites of the four countries sampled, *P. variegata* has shown to be mostly present in Spain and South Italy (Table 1), as already described for the sites El Escorial [14] and Oliveto Lucano [7, 20], as well as in a site of Central Italy where *P. variegata* has been described here for the first time (i.e. Manziana). The latter is a forest in which Turkey oak tree (*Quercus cerris*) is highly abundant, confirming the strong ecological associations between *P. variegata* and this species [8, 9]. At higher latitudes (New Forest, UK) and in the only site out of Europe (Highland Park, USA), *P. variegata* showed a narrow period of activity (Fig. 1), which might be explained by the less permissive climatic conditions observed in these sites compared to the localities in Spain and Italy (Additional file 1: Table S1). At this site in North America, the dominant tree species is the northern red oak (*Quercus rubra*), suggesting that association between oaks and the distribution of *P. variegata* may be quite general.

The present results show a direct correlation among temperature measures in the whole dataset, as well as analysing the different sites separately (Table 2), indicating that this parameter is important in the lachryphagous activity of *P. variegata* males. On the contrary, the direct correlation with mean wind speed, as well as the inverse correlation with barometric pressure and relative humidity, showed significant values only including data from all sites. But, when analysed per site, they seem to have a different relationship according to the geographical context. In fact, wind and relative humidity are negatively correlated with the lachryphagous dynamics of *P. variegata* males only in Manziana and El Escorial, respectively, indicating that their effect is probably linked to local situations and it should not be excluded that they are spurious correlations.

The few *P. variegata* females captured around human eyes in three sites out of five (i.e. Oliveto Lucano, El Escorial and Highland Park) showed correlations with day length, relative humidity and barometric pressure but not with temperature. However, even if we take this observation as anecdotal and of negligible epidemiological relevance (considering the low numbers of females collected in this study), it seems that the environmental drivers associated with females are different from those linked to males.

## Conclusions

The heterogeneity of statistical associations of climatic parameters and the different sampling points taken into account in this multicentre study indicates how complex is the population dynamics of *P. variegata* concerning its geographical distribution. Temperature is a common descriptor of *P. variegata* male lachryphagy in almost all sites (with exception of the northern area, i.e. New Forest, UK, in which however the small dataset did not allow to obtain reliable results) both at its extreme values (minimum and maximum). By sampling across a wide range of temperatures, ranging from 0 °C to 36 °C (Table 3), we were able to ascertain that the minimum temperature in which lachryphagous activity occurred ranged from 7 °C to 11 °C, regardless the latitude. If we put these results in the frame of the global climate changing scenario, we can suppose that the future increase in temperatures predicted from IPCC [21] will subsequently modify (possibly increasing) the period of activity of *P. variegata* as well as its distribution and density northwards Europe [9] and, possibly, North America. The epidemiological consequences of these results reside in the direct relationship between lachryphagous activity of *P. variegata* males and the transmission of *T. callipaeda* to the vertebrate hosts, increasing the relevance of thelaziosis in the public health that should not be neglected.

## Supplementary information

**Additional file 1: Table S1.** Monthly values of the climatic variables measured in this study per each sampled site during the period of activity of *Phortica variegata*.

## Data Availability

The datasets used and analysed during the present study are available from the corresponding author on reasonable request.

## Abbreviations

a.s.l.: above sea level
dbh: diameter at breast height
