# Supplementary material for: Temperature is a common climatic descriptor of lachryphagous activity period in Phortica variegata (Diptera: Drosophilidae) from multiple geographical locations
Source: Parasit Vectors. 2020 Feb 18;13:89. doi: 10.1186/s13071-020-3955-0 (PMC7029543; doi:10.1186/s13071-020-3955-0)
Supplement: Supplementary file 1 — Additional file 1: Table S1. Monthly values of the climatic variables measured in this study per each sampled site during the period of activity of Phortica variegata. [file 13071_2020_3955_MOESM1_ESM.docx]

**Additional file 1: Table S1.** Monthly values of the climatic variables measured in this study per each sampled site during the period of activity of *Phortica variegata*.

| Variable | Site | April | May | June | July | August | September | October | November |
| --- | --- | --- | --- | --- | --- | --- | --- | --- | --- |
| Max. temperature | Highland Park | 9.7 | 24.6 | 25.3 | 30.2 | 28.2 | 24.7 | 14.7 | 5.4 |
| (°C) | New Forest | - | 19.4 | 18.3 | 23.3 | 21.7 | 18.3 | - | - |
|  | El Escorial | 16.2 | 21.1 | 26.5 | 30.9 | 33.8 | 29.7 | 19.4 | 12.1 |
|  | Manziana | 26 | 25.9 | 30.6 | 33.9 | 32.7 | 27.6 | 25 | 21 |
|  | Oliveto Lucano | - | 25.2 | 31 | 28.5 | 30.5 | 26.4 | 21.5 | 17.5 |
| Min. temperature | Highland Park | 0.2 | 11.7 | 14.2 | 17.8 | 18.1 | 14.9 | 6.3 | -0.6 |
| (°C) | New Forest | - | 5.6 | 12.8 | 15.0 | 11.1 | 6.7 | - | - |
|  | El Escorial | 9.2 | 8.9 | 13.7 | 16.4 | 18.4 | 16.3 | 9.9 | 6.1 |
|  | Manziana | 5.3 | 9.3 | 12.6 | 14.8 | 16.3 | 15.1 | 9.4 | 5.3 |
|  | Oliveto Lucano | - | 13 | 17 | 19.5 | 19 | 15.3 | 14.7 | 14.5 |
| Mean temperature | Highland Park | 4.9 | 18.1 | 19.8 | 24.0 | 23.1 | 19.8 | 10.5 | 2.4 |
| (°C) | New Forest | - | 13.3 | 16.1 | 18.9 | 16.7 | 12.8 | - | - |
|  | El Escorial | 11.3 | 15.0 | 20.1 | 23.6 | 26.1 | 23.0 | 14.6 | 9.1 |
|  | Manziana | 14.7 | 16.3 | 19.7 | 23.6 | 23.5 | 19.9 | 16.1 | 10.3 |
|  | Oliveto Lucano | - | 19.2 | 24 | 24 | 24 | 22.5 | 19 | 16.5 |
| Wind | Highland Park | 4.7 | 3.9 | 3.5 | 3.3 | 3.3 | 3.2 | 3.9 | 4.0 |
| (m/s) | New Forest | - | 4.9 | 4.7 | 4.7 | 4.7 | 4.2 | - | - |
|  | El Escorial | 3.4 | 2.4 | 3.2 | 3.4 | 3.4 | 2.8 | 3.1 | 3.1 |
|  | Manziana | 2.8 | 2.2 | 2.4 | 2.7 | 1.9 | 1.9 | 2.7 | 2.3 |
|  | Oliveto Lucano | - | 19 | 31 | 23.5 | 22 | 12 | 36.7 | 9.5 |
| Barometric pressure | Highland Park | 995 | 996 | 994 | 998 | 996 | 1000 | 996 | 997 |
| (hPa) | New Forest | - | 1015.5 | 1016.1 | 1015.1 | 1015.2 | 1017.2 | - | - |
|  | El Escorial | 928.6 | 927.7 | 926.4 | 928.9 | 929.4 | 930.2 | 929.3 | 928.3 |
|  | Manziana | 1014 | 1012 | 1010 | 1014 | 1016 | 1016 | 1015 | 1026 |
|  | Oliveto Lucano | - | 1011.9 | 1018 | 1012.5 | 1014.1 | 1013 | 1012 | 1019 |
| Relative humidity | Highland Park | 70 | 63 | 68 | 66 | 78 | 79 | 80 | 80 |
| (%) | New Forest | - | 77 | 78 | 78 | 80 | 81 | - | - |
|  | El Escorial | 66 | 59 | 59 | 45 | 40 | 51 | 64 | 81 |
|  | Manziana | - | - | 39 | 40.3 | 60.5 | 61.6 | 65.3 | - |
|  | Oliveto Lucano | - | 64.6 | 49.3 | 54.7 | 80.3 | 79.9 | 77.1 | 71.9 |

**Data sources:**

Highland park – Data presented are for the 2018 sampling period. (Weather station KROC, Greater Rochester International Airport. National Oceanic and Atmospheric Administration, US Department of Commerce; <https://www1.ncdc.noaa.gov/pub/orders/IPS/IPS-79CE1A37-D235-4EBB-A90E-ECB7F6B6273F.pdf>)

### New Forest – Mean, maximum and minimum temperatures quoted are the mean monthly values recorded in 2018 at a local weather station (Southampton Airport, 50.95^o^N, 1.36^o^W, 17 m asl; <https://www.wunderground.com/history/daily/gb/brockenhurst/EGHH>). Wind, barometric pressure and relative humidity values are taken from HadUK-Grid Gridded Climate Observations (Met Office, 2018: HadUK-Grid Gridded Climate Observations on a 12km grid over the UK for 1862-2017. Centre for Environmental Data Analysis, <http://catalogue.ceda.ac.uk/uuid/dc2ef1e4f10144f29591c21051d99d39>). For these data, mean monthly values in a series from 2007-2017 are taken from the 12km grid square in which the sample site was located.

El Escorial - Maximum, minimum and mean temperature, wind and barometric pressure provided by the Spanish Meteorological Agency (AEMet) for the closest meteorological station (climate station: Robledo de Chavela, <http://datosclima.es> ); relative humidity: monthly average based on 2018 data (climate station: Robledo de Chavela, <http://www.meteoclimatic.net> ).

Manziana - temperature, wind, barometric pressure: monthly average based on 2018 data (climate station Canale Monterano, <http://www.arsial.it>); relative humidity: monthly average based on wheater station Ventus W928 located on the sampling site.

Oliveto Lucano - Monthly averages based on 2018 data (climate station Campomaggiore, [www.alsia.it/opencms/opencms](http://www.alsia.it/opencms/opencms)).
